# Sequence homology: A poor predictive value for profilins cross-reactivity

**DOI:** 10.1186/1476-7961-3-13

**Published:** 2005-09-10

**Authors:** Mojtaba Sankian, Abdolreza Varasteh, Nazanin Pazouki, Mahmoud Mahmoudi

**Affiliations:** 1 Immunobiochemistry Lab, Immunology Research Center, Bu-Ali Research Institute, Mashhad, Iran; 2 Molecular biology Lab, Immunology Research Center, Bu-Ali Research Institute, Mashhad, Iran

**Keywords:** food allergy, melon, profilin, cross-reactivity, epitope

## Abstract

**Background:**

Profilins are highly cross-reactive allergens which bind IgE antibodies of almost 20% of plant-allergic patients. This study is aimed at investigating cross-reactivity of melon profilin with other plant profilins and the role of the linear and conformational epitopes in human IgE cross-reactivity.

**Methods:**

Seventeen patients with melon allergy were selected based on clinical history and a positive skin prick test to melon extract. Melon profilin has been cloned and expressed in *E. coli*. The IgE binding and cross-reactivity of the recombinant profilin were measured by ELISA and inhibition ELISA. The amino acid sequence of melon profilin was compared with other profilin sequences. A combination of chemical cleavage and immunoblotting techniques were used to define the role of conformational and linear epitopes in IgE binding. Comparative modeling was used to construct three-dimensional models of profilins and to assess theoretical impact of amino acid differences on conformational structure.

**Results:**

Profilin was identified as a major IgE-binding component of melon. Alignment of amino acid sequences of melon profilin with other profilins showed the most identity with watermelon profilin. This melon profilin showed substantial cross-reactivity with the tomato, peach, grape and *Cynodon dactylon *(Bermuda grass) pollen profilins. Cantaloupe, watermelon, banana and *Poa pratensis *(Kentucky blue grass) displayed no notable inhibition. Our experiments also indicated human IgE only react with complete melon profilin. Immunoblotting analysis with rabbit polyclonal antibody shows the reaction of the antibody to the fragmented and complete melon profilin. Although, the well-known linear epitope of profilins were identical in melon and watermelon, comparison of three-dimensional models of watermelon and melon profilins indicated amino acid differences influence the electric potential and accessibility of the solvent-accessible surface of profilins that may markedly affect conformational epitopes.

**Conclusion:**

Human IgE reactivity to melon profilin strongly depends on the highly conserved conformational structure, rather than a high degree of amino acid sequence identity or even linear epitopes identity.

## Introduction

Profilins are well-known ubiquitous cytoskeleton proteins which are thought to be a link between the microfilament system and signal transduction pathways [1]. Profilin was first recognized as an allergen in birch pollen, called Bet v 2 [2]. Currently, plant profilins have been shown to be highly cross-reactive allergens that bind IgE antibodies of patients with food and tree pollen allergy [3]. Furthermore, profilins were recognized as causing allergic reaction to pear, peach, apple, melon, tomato, celery, pumpkin seeds, and peanut [4]. Several studies addressing the cross reactivity of IgE antibodies to conservative plant allergens have shown that profilins account for some of the fruit-fruit [5], fruit-plant pollen [6], and latex-food syndromes [7]. For example, it seems profilins are involved in the celery-mugwort-spice syndrome and cross-reactivity between ragweed pollen and cucurbitaceous family [8,9].

Recently, cDNA coding for a number of profilins were characterized and expressed as recombinant allergens. The tertiary structures of some of these profilins have been determined by x-ray crystallography [10]. These data provide new perspectives to the molecular basis of the cross-reactive epitopes of the profilins. Previously, we have identified, cloned and expressed melon (*Cucumis melo*) major allergen, and this allergen was introduced to the International Union of Immunological Society (IUIS) allergen nomenclature subcommittee as Cuc m 2. We observed melon-related fruits such as watermelon, cucumber and cantaloupe and found little clinical cross-reactivity with melon [11]. The aim of this study was to investigate cross-reactivity of rCuc m2 with other plant profilins and the role of the linear and conformational epitopes in these IgE cross-reactivities.

## Materials and methods

### Patient's sera

Individuals (*n = 24*) who complained of clinical symptoms after ingestion of melon were recruited at the Department of Immunology and Allergy of Ghaem Hospital Mashhad, Iran. Seventeen out of 24 patients (10 women, 7 men, mean age 34 years) were included. Diagnosis was established from clinical history and skin prick tests. The skin prick test (SPT) was performed according to the guideline of the subcommittee on skin tests of the European Academy of Allergology and Clinical Immunology [12]. The sera were collected from all of the subjects, which had a clinical history of allergic reaction to melon. A control group (*n = 15*) with no history of allergic disease and negative skin prick tests to melon was also selected.

### Allergenic extracts

After washing the fruits, the seeds were removed and the inner part of pulp isolated. Homogenized in a blender and extracted in phosphate-buffer (1:10 w/v) 100 mM (pH 8.2) containing 1% (w/v) polyvinyl pyrrolidone, 10 mM ethelene diaminetetraceticacid (EDTA), and 10 mM diethyldithiocarbamate (DIECA). The slurry was centrifuged (15000 g) for 30 min at 4°C and fractionated in the range of 30% to 60% saturation of (NH4)_2_SO4 to enrich melon profilin. The pellet was dissolved and extensively dialyzed against phosphate-buffer 100 mM pH 7.4 (4°C, 72 h) and freeze-dried. Some of the lyophilized samples were reconstituted in distilled water (1/10 w/v) and glycerinated for skin testing. *Cynodon dactylon *(Bermuda grass) and *Poa pratensis *(Kentucky blue grass) pollens (Sigma) were extracted as described previously [13]. Allergen extract of kiwi and banana were prepared as described previously by Moller et al [14]. Presence of profilin in all of the extraction was proven by immunoblot analysis using peroxidase conjugated rabbit polyclonal antibody against saffron pollen profilin (kindly provide by F. Shirazi, Bu-Ali Research Institute, Mashhad, Iran) (data not shown).

### Cloning, expression and purification of rCuc m 2

Total RNA was extracted from 1 g of fine powder from melon pulp grounded under liquid nitrogen by means of the Concert™ plant RNA purification kit (Invitrogen). First-strand cDNA was synthesized from 2 μg total RNA using a first-strand cDNA synthesis Kit (Fermentas) with a Oligo (dT)_18 _as primer. The Cuc m 2 coding region was amplified with Pfu DNA polymerase (Fermentas), using two specific primers. According to the sequence of Cuc m 2 (GenBank accession number: AY271295), the 5' primer (5'-TCA*CATATG*TCGTGGCAAGTTTACGTCG-3') mimics the first six codons and introduces an *NdeI *restriction site (underlined). The 3' primer (5'-AAG*CTCGAG*GCCCTGATCAATAAGATAATC-3') mimics the last seven codons, excluding the stop translation codon, and introduces an *Xho I *restriction site (underlined). After PCR amplification, the 400-bp product was ligated into pET21b^+ ^(Novagen). The fidelity of the cloned product was verified by sequencing. The resulting pET21b^+^/Cuc m 2 construct was transformed into BL21 (DE3) strain of *Escherichia coli*. Expression and purification of rCuc m 2 were carried out as described previously [15]. Purified rCuc m 2 was then subjected to reducing SDS-PAGE, and eletroblotted on PVDF membrane.

### rCuc m 2-specific IgE an inhibition ELISA

The wells of the ELISA microplate (Nalgen Nunc International) were coated with 100 μl of recombinant melon profilin (rCuc m 2) at a concentration of 50 ng/well in coating buffer (15 mM Na2CO3 and 35 mM NaHCO3, pH 9.6) at 4°C for 16 h. After blocking with 150 μl of 2% BSA in PBS at 37°C for 30 min., the plates were incubated with 100 μl of patients sera for three hours at RT followed by incubating with a goat biotinylated anti-human IgE (KirKeggard & Perry laboratories) diluted 1/1000 in PBS containing 1% BSA for 2 hours. The wells were then incubated for 1 h with strepavidin horseradish peroxidase-labeled (Sigma) diluted 1/1000 in PBS containing 1% BSA. Each incubation step was followed by 5 washes with PBS-T (PBS containing 0.05% Tween 20). Enzyme reaction was performed using tetramethyl benzidine (TMB)/H_2_O_2 _as the substrate. The reaction was stopped by 3 M HCl after 30 minutes at RT in darkness and the absorbance was read at 450 nm. Results were expressed as optical density (OD) units. Based on the mean value of 15 normal sera (<0.3 OD unites), OD value of greater than 0.6 were considered positive.

In order to assess relatedness of rCuc m 2 to profilins from other fruit and pollen, ELISA inhibition was carried out as follows: 100 μl of a pooled serum comprising five sera from subjects showing IgE antibodies to rCuc m 2 preincubated with 100 μl of different concentrations of extracts of *Cynodon dactylon *(Bermuda grass) and *poa pratensis *(Kentucky blue grass) pollen, melon, watermelon, banana, peach, cantaloupe, tomato and grape, rCuc m 2 and BSA for 2 hours at room temperature. This solution was then added to a flat-bottomed microtiter plate that had been coated with rCuc m 2 (50 ng/well). The ELISA procedure thereafter was the same as described for measurement of melon allergen-specific IgE.

#### SDS-PAGE and immunoblotting analysis

Sodium dodecylsulfate polyacrylamide gel electrophoresis (SDS-PAGE) of melon extract and rCuc m 2 was performed according to laemmli [16] using a separation gel of 15% acrylamide under reducing conditions. Separated protein bands were electro-transferred to polyvinylidene difluoride (PVDF) membranes (Immobilon P, Millipore Corp., Bedford, MA, U.S.A.), essentially by the method of Towbin *et al *[17].

Immunodetection was carried out on PVDF after treatment with methanol for 15 sec and blocking with Superblock at 4°C for 16 h. Membranes were probed with individual sera from melon-allergic patients (diluted 1/5 in PBS containing 1:10 v/v blocking buffer) or with sera from non-allergic subjects for 4 h (overnight for IgE immunoblot of total extract) at room temperature. Stripes are then washed 4 times for 5 min with 0.05% Tween-20 in PBS and incubated for 2 h with a rabbit anti-human IgE polyclonal antibody conjugated with peroxidase (DAKO) diluted 1/2000 in PBS containing blocking buffer (1:10 v/v). After washing, the peroxidase reaction was developed with Super Signal West Pico Chemiluminescent substrate (Pierce) for 5 min, and IgE-binding proteins were detected by ECL-hyperfilm (Amersham Pharmacia Biotech) after exposure for 1 min.

### Fragmentation of rCuc m 2 and immunoblotting analysis

To investigate the role of the linear and conformational epitopes in the IgE binding to the rCuc m 2, a combination of chemical cleavage and immunoblotting techniques was used. Amino acid sequence analysis of Cuc m 2 revealed an Asp-Pro site at the position of 57–58 that makes it susceptible to cleavage by pH 2.5. Partial acid hydrolysis was carried out according to the protocol described by Inglis [18]. Briefly, 50 μl of rCuc m 2 (1 mg/ml) was added to 150 μl formic acid and incubated for 24–48 h at 37°C and room temperature. The resulting fragments were separated by tricine-SDS-polyacrylamide gel and visualized by silver staining [19]. Immunodetection of separated protein bands was carried as described above. This immunoblotting analysis was performed with a pooled serum from five melon allergic patients (No: 2, 6, 8, 13 and 14) that showed IgE immunoblot reactivity with 14.5 kDa component of melon extract and r Cuc m 2.

Alternatively, the membrane was blocked with 5% skim milk and incubated with a peroxidase conjugated rabbit polyclonal antibody against saffron pollen profilin at a 1:1000 dilution in PBS containing 2.5% skim milk. The peroxidase reaction was developed as described above.

### Structure prediction and modelling

The deduced amino acids sequence of Cuc m 2 was subjected to a BLAST similarity search. A mulitple alignment of the homologous allergens sequences was performed by BioEdit and modified manually when necessary [20]. The percentage identities were determined by comparison of the amino acid sequences after multiple sequence alignment (Fig. 3).

Solvent accessibility and charge distribution of an antigen surface may play prominent roles in immunoreactivity of a epitope. Therefore, in order to display the theoretical effect of amino acid differences between rCuc m 2 and other profilins on the solvent accessibility and charge distribution of the rCuc m 2 surface area, comparative models of tomato, watermelon and melon profilins were generated using the Internet server Swiss Model . The profilin models were built using the X-ray structure of 1g5uB as template. This protein has, respectively, 77.9%, 82.4% and 74.8% sequence identity with melon, tomato and watermelon profilin. The program ZMM was then used with the above constraints to minimize the conformational energy of the proteins [21]. The ZMM uses the Amber all-atom force field [22]. The AMBER force field with a cut-off distance of 8 Å has been used to minimize conformational energy in the space of generalized coordinates including torsion and bond angles. Low-energy conformations were searched by the Monte Carlo minimization method [23]. Monte Carlo trajectories were terminated when 500 sequential energy minimizations did not improve the lowest-energy conformation. Calculations and analysis of low-energy conformers were performed using the ZMM molecular modeling package. The essential accuracy and correctness of the models were evaluated using PROCHECK and WHAT-IF program from online Biotech Validation Suite . All molecular models were viewed and examined for accessible and electrostatic energy of the protein surface using the Swiss Pdb Viewer program. We have ignored solvating effects and used Coulomb law for the calculations of the electrostatic energy.

## Results

The seventeen patients suffering from melon allergy were included in our study. Case histories in respect to melon allergy are summarized in Table 1. Oral allergy syndrom and rhinoconjunctivitis were the most prominent manifestations on ingesting melon (94 and 58%, respectively). Sera from 11 of 17 (64%) patients showed increased IgE reactivity to rCuc m 2. Therefore, the melon profilin, rCuc m 2, was identified as a major allergen. Melon allergic individuals also showed clinical features of allergic reaction to fruit from various botanical families such as grape (58%) and tomato (35%).

**Table 1:** Clinical data, rCuc m 2-specific IgE levels and SPT responses of the selected patients with allergy to melon

Patient No.	Age (years)	Sex	Symptoms*	Allergy to other fruits	rCuc m 2 Specific IgE (OD^ξ^)	SPT with melon extract (mm)
1	29	F	R	Grape, Kiwi	0.68	5
2#	52	M	RC, OAS, D, U, G	Grape	0.97	12
3	24	F	RC, OAS	Tomato	0.72	5
4	30	M	RC, E, SI, OAS, U	Grape	0.84	8
5	31	M	OAS, C	Kiwi,Tomato	0.63	5
6#	28	M	RC, OAS, SI	Tomato, grape, peach, zucchini, cantaloupe,	1.2	8
7	44	M	RC, OAS, SI, G	Cantaloupe, Kiwi	0.65	5
8#	43	F	RC, OAS, SI, C	Walnut, Spice	1.02	8
9	30	F	R, OAS	Grape	<0.3	4
10	24	F	RC, OAS, SI, C	Grape, Tomato, zucchini, Cantaloupe	1.32	4
11	46	M	R, OAS, C, D	ND	<0.3	5
12	39	F	RC, OAS, U, SI	Grape, Tomato	<0.3	3
13#	28	F	RC, OAS, SI, C, E	Tomato	0.83	10
14#	27	F	R, OAS	Fig, grape, zucchini	0.94	5
15	45	F	OAS	Zucchini, watermelon	<0.3	4
16	21	M	R, OAS	Zucchini, grape, watermelon	<0.3	8

17	39	F	RC, OAS, U, SI, D	Grape, garlic	<0.3	10

* *C*, cough; *D*, dyspnea; *E*, eczema; *R*, rhinitis; *RC*, rhinoconjunctivitis; *G*, *g*astrointestinal symptoms; *SI*, Skin itching; *U*, urticaria; *OAS*, Oral allergy syndrome (OAS; defined as the onset of immediate oral itching with or without angioedema of the lips and oral mucosa); *ND*, not determined. #; Patients' sera were selected for inhibition assays. ξ; OD, Optical density.

**Figure 1:**
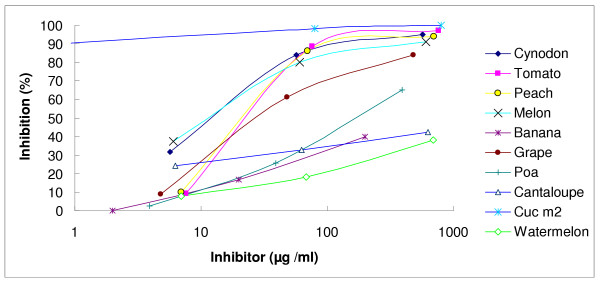
Inhibition of the binding of IgE antibodies in sensitized serum to immobilized rCuc m 2. Inhibition was assayed by a competitive ELISA method. The pooled sera (1: 5 dilution) was preincubated for 1 h with an equal volume of various concentrations from each extraction solution which was made in PBS before adding to the plate coated with rCuc m 2 (50 ng/well). Sample concentrations are expressed as those in preincubation mixture. Inhibition with BSA was used as negative control (not shown).

Sera from patients no; 2–4, 6, 8,13 and 14 that indicated highest level of specific IgE against rCuc m 2 in ELISA were selected for melon extract immunoblotting. Patients' sera no. 2, 6, 8, 13 and 14 (Table 1) reacted only with the 14.5 kDa component of melon extract (Data not shown). To prepare the inhibition assay pool of sera, reactivity of all of these sera with melon profilin was confirmed by a positive IgE immunoblot reactivity to rCuc m 2. Inhibitions of IgE binding to rCuc m 2 by other plant profilins are represented in Fig. 1. All of the melon, Bermuda grass pollen, peach, tomato and grape extracts revealed significant inhibition of IgE binding to rCuc m 2, and cantaloupe extract showed less significant inhibition. In contrast, watermelon, banana and *poa pratensis *indicated no notable inhibition.

**Figure 2:**
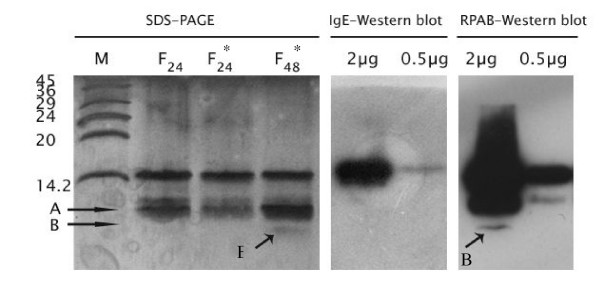
SDS-PAGE and immunoblot analysis of acid hydrolyzed rCuc m2. silver stained-SDS gel electrophoresis of rCuc m2 after incubation with 75% formic acid for 24 h and 48 h at room temperature (F_**24**_) and 37°C (F*_24 _and F*_48_). "A" and "B" arrow indicate protein band with molecular mass of approximately 6 and 9 kDa. Figure in the middle shows IgE-immunoblotting of two concentration of F*_48 _using a pooled serum of melon profilin-sensitized individuals (in the middle) and figure on the right display immunoblotting of the same sample with rabbit polyclonal anti-saffron antibody.

The best result for acid hydrolysis was achieved by 24 hours incubation at 37°C. The fragments of acid hydrolyzed rCuc m 2 were resolved into two distinct bands (10 and 6 kDa). In addition, a 14.5 kDa protein band appeared as a complete rCuc m 2 molecule that was not affected by acid hydrolysis (Fig. 2, at the left). The hydrolyzed rCuc m 2 was assessed with the rabbit polyclonal antibody against saffron pollen profilin and a pooled serum of five melon-sensitive individuals in immunoblotting analysis. Immunoblotting analysis with rabbit polyclonal antibody shows the reaction of the antibody to the 14.5, 9, and 6 kDa protein bands (Fig. 2, on the right). In contrast, IgE-blotting displayed only a major IgE-binding band at approximately 14.5 kDa to which pooled serum reacted (Fig. 2, in the middle).

**Figure 3:**
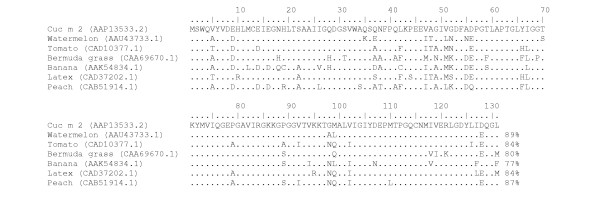
Comparison of Cuc m 2 with different plant profilins, including watermelon (*Citrullus lanatus*), tomato (*Lycopersicon esculentum*), Bermuda grass (*Cynodon dactylon*), banana (*Musa acuminate*), peach (*Prunus persica*) and latex (*Hevea brasiliensi*). Amino acid sequence identity of Cuc m 2 with other members of profilin family are indicated at the end of each amino acid sequence. Areas covering experimentally determined sequential IgE-reactive epitopes are underlined.

The deduced protein sequence of Cuc m 2 was subjected to a BLAST similarity search that showed the highest degrees of identity with profilins from the following sources: *Citrullus lanatus *(Watermelon)*, Ricinus communis *(Castor bean)*, Phaseolus vulgaris *(Green bean), *Hevea brasiliensis *(Latex), *Lycopersicon esculentum *(Tomato), *Capsicum annuum *(Pepper), *Prunus persica *(Peach), *Cynodon dactylon *(Bermuda grass), respectively. Figure 3 shows an alignment of the Cuc m 2 amino acid sequence with profilins of other plants.

**Figure 4:**
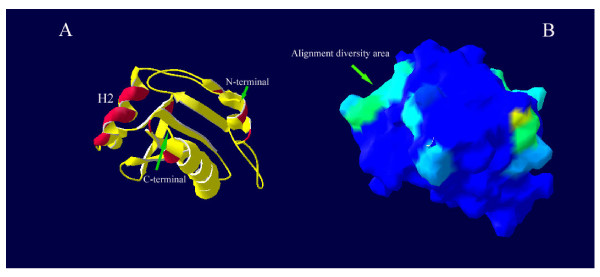
Three-dimensional view of melon profilin model. (A) Amino acid differences with watermelon profilin indicated in red on the ribbon diagram of Cuc m 2 model, H2 shows second α-helix. (B) Most of these amino acid differences located at the solvent accessible area of the Cuc m 2 surface and displayed in light blue color.

**Figure 5:**
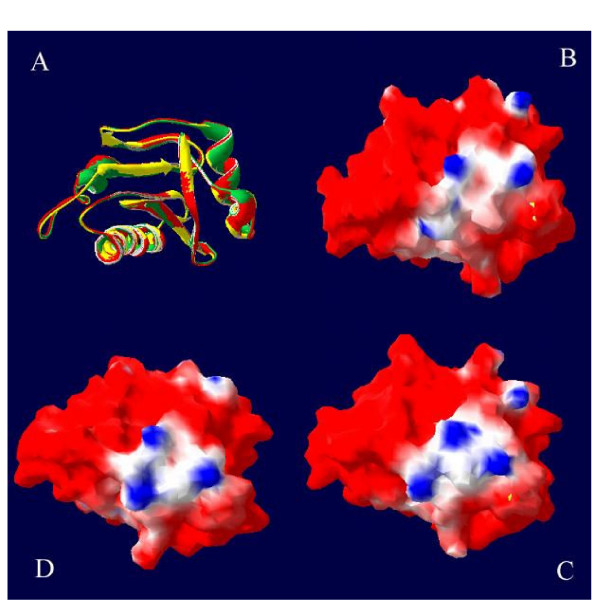
(A) Superimposing of tomato (red ribbon), watermelon (green ribbon) and melon (yellow ribbon) profilin models. Electrostatic potentials at the surface of melon (B) watermelon (C) and tomato (D) profilin models. Blue represents positive potentials and red represents negative potentials. Orientations of B, C and D models are the same as in part A.

Three-dimensional structure of the tomato, watermelon and melon profilins are shown in figure 4 and 5. The models were evaluated in terms of stereochemical and geometric parameters such as bond lengths, bond angles, torsion angles, G-factor and packing environment, and they were found to satisfy all stereochemical and geometric criteria. No residue was located in the disallowed regions of the Ramachandran map. After energy minimization of the models, the overall conformational energy of comparative models of tomato, watermelon and melon profilins are -765, -792 and -709 kcal/mol, respectively. Main-chain Cα atoms of 1g5uB, melon, watermelon and tomato profilin superimpose with an RMS deviation of 0.80, 0.77 and 0.82 Å, respectively. Superimposing of the 3-dimensional models of the melon, watermelon, tomato and latex profilin (1g5uB) showed nearly the same tertiary structure. Alignment of the three-dimensional model of watermelon and melon indicated most of the alignment diversity located on the accessible area of watermelon profilin (Fig. 4). In addition to accessible area of molecule surface, amino acid differences among profilins influence – the electric potential of the solvent-accessible surface of profilins (Fig. 5).

## Discussion

Allergen immunotherapy and diagnosis rely on the use of high quality natural allergenic products. However, apart from improved standardization and quality control, there have been few significant innovations in allergen immunotherapy in recent years. In the last decade, There has been remarkable progress in the molecular biology of allergens and more than a hundred food allergens have been cloned and expressed in the prokaryotic, yeast and eukaryotic expression systems. More over, classification of allergens in to the groups based on similarity provides an optimistic prospective to diagnosis and treatment with a small panel of cross-reactive allergens which reflect a high number of allergens. The cross-reactivity of allergens has to be well characterized to define panels of cross-reactive allergens, the pattern of clinical sensitivities and the probability of novel foods being allergen [24,25].

Several studies have focused on establishing the actual patterns of allergen cross-reactivity. In some cases high sequence homology is related to pan-allergenicity as in lipid transfer proteins [26], while in other cases high homology does not result in cross reactivity as in birch and carrot cyclophilins [27]. In this study, we aimed to define cross-reactivity rules in profilins. The result of a sequence homology search reveals high similarity among profilins. Despite high sequence similarity among profilins, our study indicated that high homology between two profilins does not necessarily results in their cross reactivity. Alignment of amino acid sequences of Cuc m 2 and watermelon showed up to 89 percent identity (Fig 3). However, there were only two patients with history of allergy to watermelon in 17 allergic individuals to melon (Table 1). This lack of clinical cross-reactivity between melon and watermelon was confirmed by inhibition experiments (Fig. 1). Although the profilin sequence of cantaloupe, the other fruit belonging to the same family as melon is not available, it seems that only a little cross reactivity can be found between these two according to our results (Fig. 1, Table 1). Interestingly, extracts of peach, tomato, grape and *Cynodon dactylon *inhibit IgE binding to Cuc m 2 nearly the same as melon extract. The rCuc m 2 showed lower identity with profilins of these plants than with the watermelon profilin. According to continuous epitope mapping and structural analysis of birch and sunflower pollen profilins, the amino acid composition of each B cell epitope was located at the 1–7, 39–46, 98–107 and 105–114 positions [28-30]. Comparison of melon and watermelon profilin amino acid sequences revealed no significant differences at the continuous epitope sites (Fig. 3). Therefore, it would be advisable to assess if conformational epitopes are involved in IgE binding to rCuc m 2. In order to define the role of the continuous and discontinuous epitopes in IgE binding to melon profilin, we used a combination of chemical cleavage and immunoblotting techniques. Cleavage of melon profilin into two fragments destroyed human IgE binding of both fragments and only whole Cuc m 2 showed IgE-binding activity. In contrast, rabbit polyclonal anti-Cuc m 2 showed similar binding activity to Cuc m 2 fragments (Fig. 2). It seems rabbit polyclonal antibody and human IgE recognize distinct epitopes on the profilin molecules. These experiments confirmed findings that indicated sunflower pollens and melon profilins lost their reactivity with the pooled sera of patients with melon allergy after treatment with pepsin [31]. The study of Rihs *et al*. also demonstrated that only the full-length soybean profilin was able to bind with IgE antibodies and any of the three overlapping recombinant fragments of soybean profilin comprising amino acid residues 1–65, 38–88, and 50–131 did not show significant binding reactivity [32].

We used 3D structural modelling to construct models of profilin allergens and explain these results. Most of amino acid differences between watermelon and melon profilins were located at the accessible site of α-Helix (especially H2) and β-turns (Fig. 4). It seems that these residues dramatically alter solvent accessibility (Fig. 4) and the electric potential of the protein surface area (Fig. 5). Both could result in changes in IgE-binding capacity of conformational epitopes, despite similar folding patterns of the plant profilins. This is mainly due to this fact that protein folding is liberal with respect to amino acid substitutions for many positions in the sequence. Such substitutions may markedly affect the protein outer surface or directly involve contact residues important for the antigen-antibody interaction, thus reducing or abolishing antibody reactivity [33]. Fortunately, these alterations will not always influence IgE binding activity of an epitope. Nuclear magnetic resonance studies indicate that only a small number of residues within an epitope are functionally important for antibody binding [34]. It could be the reason for melon profilin cross-reactivity with tomato, peach, *Cynodon dactylon *and grape profilins. This evidence led to the suggestion that a shared topology and conformational epitope is the presumed basis for extensive IgE cross-reactivity between Cuc m 2 and other plant profilins. On the other hand, earlier studies on Cuc m 2 oligomerization showed multimer forms of Cuc m 2 had more IgE activity than monomer Cuc m 2 (data not published). If we assume that polymerization patterns of profilins are similar to human profilin [35], most of the reported sequential epitopes will be located at the inaccessible site of multimeric profilins.

In conclusion, The presence of IgE cross-reactivity among profilins strongly depends on the highly conserved conformational structure, rather than the percentage of amino acid sequence identity. Clarifying conformational and sequential epitopes of profilin may open up novel ways to improve our knowledge about cross-reactivity among profilins. It would be useful to define cross-reactive clusters of profilin and other allergen molecule families in order to reach a diagnosis and treatment strategy based on a small set of cross-reactive allergens.
